# Supplementary material for: The olfactory gating of visual preferences to human skin and visible spectra in mosquitoes
Source: Nat Commun. 2022 Feb 4;13:555. doi: 10.1038/s41467-022-28195-x (PMC8816903; doi:10.1038/s41467-022-28195-x)
Supplement: Supplementary file 1 — Supplementary Information [file 41467_2022_28195_MOESM1_ESM.pdf]

Supplementary Information Tables, Figures and Legends

Supplementary Information Table 1

$Op1^{R}$	5′-GGTTCGGAGGCTTCGAGTACCGG-3′
$Op2^{G}$	5′-TGGAGGGAGTGCGGAGGCTTGGG-3′

The target sequences of the sgRNAs used for gene targeting. The PAM sequences are underlined.

Supplementary Information Table 2

$Op1^{R}$	Upstream homology arm:<br>F: 5′-GCGCACATTTCCTTATCATGCAGTCCAGTCTGTCT-3′;<br>R: 5′-TCTTAACGCGAGTTAGAAGATGTAGATCACGCAAC-3′;<br><br>Downstream homology arm:<br>F: 5′-ATCGATAAGCGCTAGGAAGCCTCCGAACCCCATCC-3′;<br>R: 5′-GATTTCATTCGCTAGGTTTGCCATCATTAATATCA-3′;
$Op2^{G}$	Upstream homology arm:<br>F: 5′-GCGCACATTTCCTTACGCCTTGCACCGAGATTTTA-3′;<br>R: 5′-TCTTAACGCGAGTTATGTGCTGGTGAAGATT-3′;<br><br>Downstream homology arm:<br>F: 5′-ATCGATAAGCGCTAGCGCACTCCCTCCAACCTG-3′;<br>R: 5′-GATTTCATTCGCTAGAACACCACAGCCATTTCGGA-3′;

PCR primers used for cloning homology arms. F and R are the forward and reverse primers, respectively.

Supplementary Information Table 3

Op1<br><br>($op1^{R}$)	F: 5′-ACCGCAAGCAACACTTTACG-3′;<br><br>R: 5′-CAAGCGATTTATATTTAGAT-3′;
Op2<br><br>($Op2^{G}$)	F: 5′-AAGTTATCAGCAAAAGTATC-3′;<br><br>R: 5′-ACCGTGTACGTTCTGTAGCG-3′;

PCR primers used for genotyping. F and R are the forward and reverse primers, respectively.

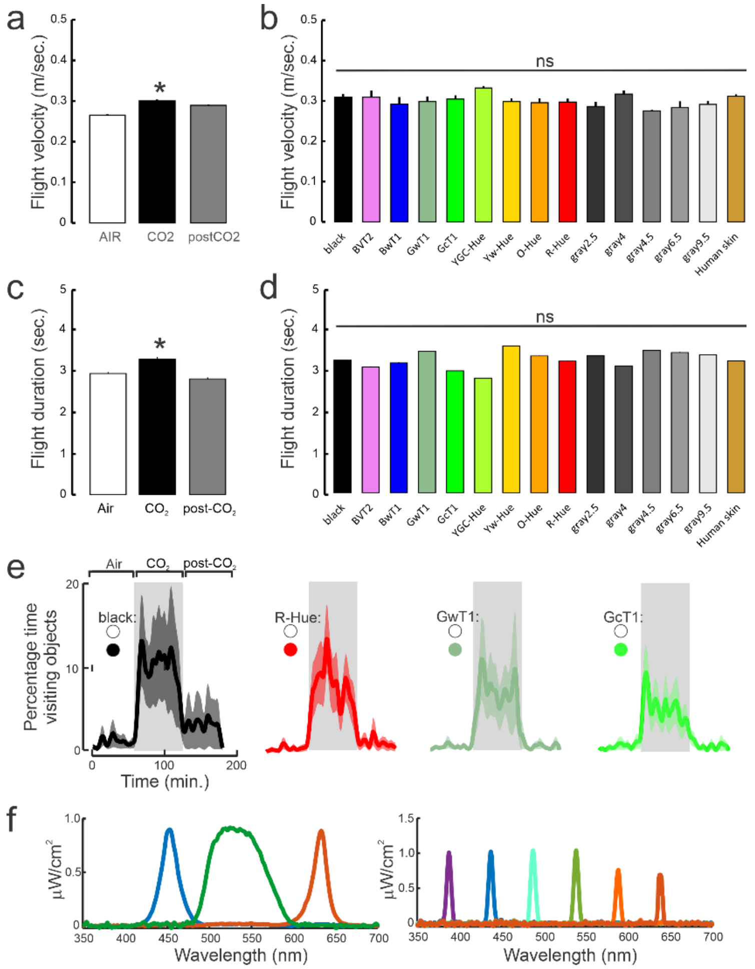

Figure S1. Flight behaviors to different color stimuli, and characterization of stimuli in ERG experiments. (a) Flight velocities of mosquitoes in different CO_2 exposure treatments (AIR-, +CO_2, and post-CO_2), across all color stimuli. Exposure to CO_2 significantly elevated the flight velocities of mosquitoes (Kruskal-Wallis test with multiple comparisons: df=2, Chi-sq. = 597.23, P < 0.001), although there was no significant difference between AIR and post-CO_2 treatments (Kruskal-Wallis test with multiple comparisons: P>0.05). Bars are the mean ± sem. (**b**) The flight velocities for each tested hue. There was no significant difference between wavelength treatment groups (Kruskal-Wallis test: df=11, Chi-sq. = 10.17, P = 0.42). Bars are the mean ± sem. (**c**) As in a, except for the mosquito flight durations. Exposure to CO_2 significantly increases the duration of the flight trajectories (Kruskal-Wallis test with multiple comparisons: df=2, Chi-sq. = 87.83, P < 0.001), although there was no significant difference between AIR and post-CO_2 treatments (Kruskal-Wallis test with multiple comparisons: P>0.05). (**d**) As in b, except for the flight durations of each visual stimulus. There was no significant difference between dominant wavelength treatment groups (Kruskal-Wallis test: df=11, Chi-sq. = 16.82, P = 0.16). Histograms in panels (b) and (d) represent n = 53,786; 23,694; 34,343; 31,037; 32,257; 24,774; 42,595; 20,929; 48,198; 25,896; 36,050; 28,644; 37,085; 27,537; and 91,674 mosquito trajectories for the black-and-white, Bv-T2, Bw-, Gw-T1, Gc-, YGc-, Yw- , O-, R-Hue, grey2.5, grey4.0, grey4.5, grey6.5, grey9.5 and faux human skin treatments, respectively. (**e**) The number of mosquitoes visiting the visual objects over the duration of the experiment. Few mosquitoes investigated the visual objects before or after the CO_2 exposure (shaded grey area denotes CO_2), and there was no significant difference between those two time periods (Kruskal-Wallis test with multiple comparisons between AIR and post-CO_2: P > 0.98). However, exposure to CO_2 significantly increased the numbers of mosquitoes visiting the visual objects (Kruskal-Wallis test with multiple comparisons: df=11, Chi-sq. = 258.72, P < 0.001). During CO_2, there were no significant differences in the number of mosquitoes investigating the different objects (Kruskal-Wallis test with multiple comparisons: P > 0.99). Lines are the means and shaded areas the ±sem. (**f**) The intensity measurements of stimuli used in the ERG experiments. Experiments used either a short-throw projector (left) or a digital monochromator (right).

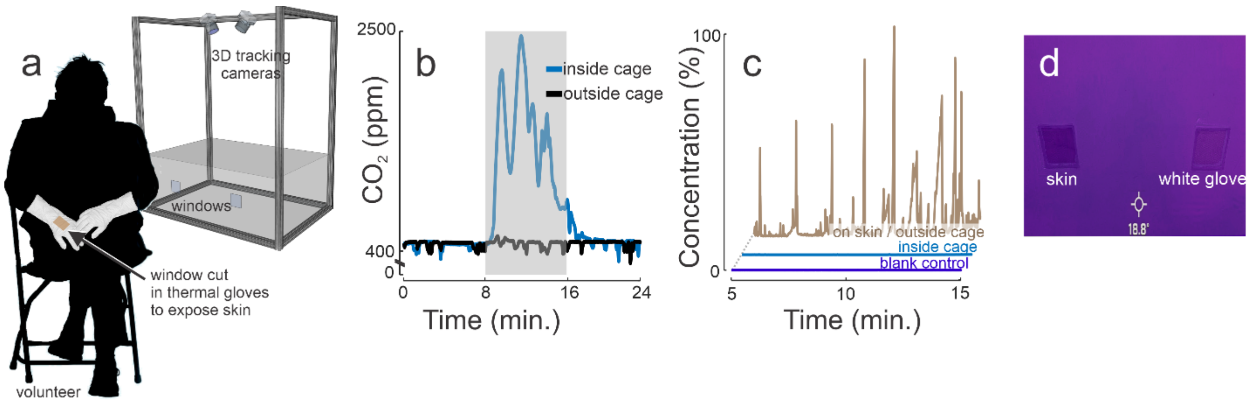

Figure S2. CO_2, skin volatiles,and temperature measurements in the cage assay. (**a**) The experimental set-up for assaying reflected spectral information from skin from volunteers. Individuals wore two white thermal gloves: one a control, and another with a window cut-out to expose the skin. (**b**) CO_2 measurements were taken outside (black trace) and inside (blue trace) the cage during experiments to measure if background contamination was occurring. CO_2 concentrations only increased in the cage during pre-programmed release from the mass flow controllers (grey shaded area). (**c**) Solid-phase microextraction fibers were used to characterize the potential for contamination from human (*lato sensu* skin) volatiles. Measurements were taken immediately adjacent to skin (brown trace), inside the cage (blue trace), and a blank control (dark blue). The VOC profiles and emission levels were not different from SPME fibers from inside the cage and the blank control. See Methods section for more details. (**d**) Image taken from a FLIR camera (FLIR One Pro, FLIR Systems Inc., Goleta, CA USA) showed a constant temperature range, and did not show any variability including the region of the cage where the volunteer's skin was displayed through the window. The thermal shielding and IR absorptive windows in the cage prevented a radiant heat signature that is attractive to mosquitoes.

a

	Coe. est.	Std. error	t-value	p-value
intercept	-1.759	0.4417	-3.983	0.0002
contrast	0.014	0.0039	3.705	0.0004
peak	0.001	0.0004	3.859	0.0003

Residential Std. error = 0.22, df = 61, Adj. R-sq = 0.23
F =10.43, df = 2 and 61, P < 0.001

b

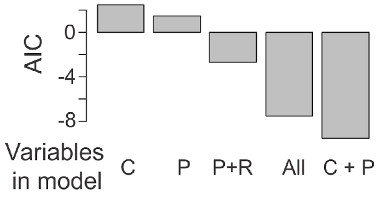

c

	Coe. est.	Std. error	t-value	p-value
intercept	0.219	0.028	7.822	<0.0001
PC2	0.002	0.001	3.46	0.001
PC3	0.002	0.001	1.729	0.089

Residential Std. error = 0.22, df = 61, Adj. R-sq = 0.17
F = 7.481, df = 2 and 61, P = 0.001

d

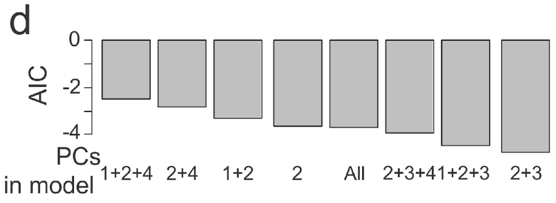

e

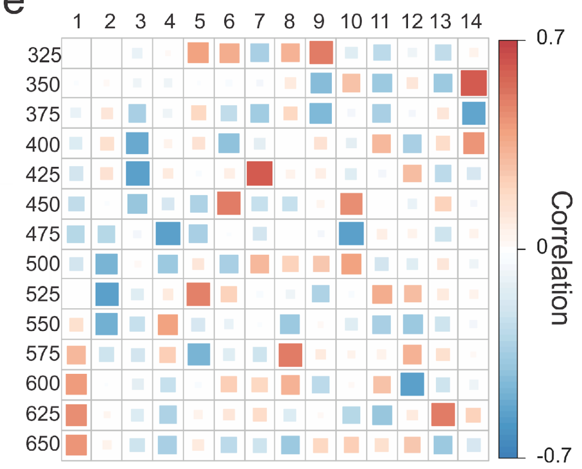

Figure S3. Models on the contributions of contrast and hue wavelength on mosquito preferences. (**a**) Linear model results based on contrast and peak wavelength values using linear regression two-tailed t-test to sample data. Bonferroni corrections were made for multiple comparisons. (**b**) AIC of all the models significantly different from a null model and based on combinations of the contrast (C), peak wavelength (P) and brightness (R) values. The model in (a) was selected as the best having the lowest AIC values. (**c**) From the linear regression two-tailed t-test to sample data, linear regression parameters, standard errors, t-values and P-values for the model based on the PC2 and PC3. The PCs were obtained from the area-under-the-curve (AUC) of the reflectance curves calculated every 25 nm from 325 to 675 nm. Bonferroni corrections were made for multiple comparisons. (**d**) AIC of all the models significantly different from a null model and based on combinations of the first 4 PCs (representing more than 99% of the variance of the original dataset). The model in (c) was selected as the best having the lowest AIC values. (**e**) Correlation matrix between the AUC values from 325 to 675 nm and the 14 resulting PCs. The first three PCs correlated with different parts of the spectrum: PC1 was strongly positively correlated with the 575-675 nm range whereas PC2 and PC2 strongly negatively correlated with the 475-575 nm and 375-475 nm range respectively.
