# Supplementary material for: The olfactory gating of visual preferences to human skin and visible spectra in mosquitoes
Source: Nat Commun. 2022 Feb 4;13:555. doi: 10.1038/s41467-022-28195-x (PMC8816903; doi:10.1038/s41467-022-28195-x)
Supplement: Supplementary file 3 — Reporting Summary [file 41467_2022_28195_MOESM3_ESM.pdf]

nature portfolio

Corresponding author(s): Jeff Riffell, at jriffell@uw.edu

Last updated by author(s): Jul 28, 2021

Reporting Summary

Nature Portfolio wishes to improve the reproducibility of the work that we publish. This form provides structure for consistency and transparency in reporting. For further information on Nature Portfolio policies, see our Editorial Policies and the Editorial Policy Checklist.

Statistics

For all statistical analyses, confirm that the following items are present in the figure legend, table legend, main text, or Methods section.

n/a	Confirmed	
☐	☒	The exact sample size (n) for each experimental group/condition, given as a discrete number and unit of measurement
☒	☐	A statement on whether measurements were taken from distinct samples or whether the same sample was measured repeatedly
☐	☒	The statistical test(s) used AND whether they are one- or two-sided<br>*Only common tests should be described solely by name; describe more complex techniques in the Methods section.*
☐	☒	A description of all covariates tested
☐	☒	A description of any assumptions or corrections, such as tests of normality and adjustment for multiple comparisons
☐	☒	A full description of the statistical parameters including central tendency (e.g. means) or other basic estimates (e.g. regression coefficient) AND variation (e.g. standard deviation) or associated estimates of uncertainty (e.g. confidence intervals)
☐	☒	For null hypothesis testing, the test statistic (e.g. F, t, r) with confidence intervals, effect sizes, degrees of freedom and P value noted<br>*Give P values as exact values whenever suitable.*
☒	☐	For Bayesian analysis, information on the choice of priors and Markov chain Monte Carlo settings
☒	☐	For hierarchical and complex designs, identification of the appropriate level for tests and full reporting of outcomes
☒	☐	Estimates of effect sizes (e.g. Cohen's d, Pearson's r), indicating how they were calculated

Our web collection on statistics for biologists contains articles on many of the points above.

Software and code

Policy information about availability of computer code

Data collection	This information is included in the manuscript (page 27)
Data analysis	This information is included in the manuscript (page 27)

For manuscripts utilizing custom algorithms or software that are central to the research but not yet described in published literature, software must be made available to editors and reviewers. We strongly encourage code deposition in a community repository (e.g. GitHub). See the Nature Portfolio guidelines for submitting code & software for further information.

Data

Policy information about availability of data

All manuscripts must include a data availability statement. This statement should provide the following information, where applicable:

- Accession codes, unique identifiers, or web links for publicly available datasets
- A description of any restrictions on data availability
- For clinical datasets or third party data, please ensure that the statement adheres to our policy

This information is included in the manuscript (page 27)

Field-specific reporting

Please select the one below that is the best fit for your research. If you are not sure, read the appropriate sections before making your selection.

☒ Life sciences ☐ Behavioural & social sciences ☐ Ecological, evolutionary & environmental sciences

For a reference copy of the document with all sections, see nature.com/documents/nr-reporting-summary-flat.pdf

Life sciences study design

All studies must disclose on these points even when the disclosure is negative.

Sample size	Each visual stimulus was tested in 4 to 12 experimental trials (on average, approximately 5,575 trajectories were quantified per trial) - this provided enough power to statistically examine attraction to a visual object.
Data exclusions	For experiments using wildtype mosquito lines (Aedes aegypti: Rockefeller, Liverpool; Anopheles stephensi (Indian strain) and Culex quinquefasciatus) and Aedes aegypti lines with the ability to detect carbon dioxide (opsin-1, opsin-2, and opsin-1,opsin-2 lines), if the mosquitoes did not respond to CO_2 during the experiment, then the trials were not used. This happened rarely, and only associated with problems with the CO_2 delivery system.
Replication	Experimental replicates were successfully performed and repeated over the course of 24 months.
Randomization	The position of the visual objects in each replicate trial was randomized to control for positional effects.
Blinding	During data collection, investigators were not blinded to the visual treatments used in the assays, although investigators were blinded to assays using the mosquito mutant lines (Gr3[ECFP], op-1, op-2, and op-1,op-2 lines) and associated wildtype controls as a control for any subtle differences in behavior between opsin mosquito lines.

Reporting for specific materials, systems and methods

We require information from authors about some types of materials, experimental systems and methods used in many studies. Here, indicate whether each material, system or method listed is relevant to your study. If you are not sure if a list item applies to your research, read the appropriate section before selecting a response.

Materials & experimental systems

n/a	Involved in the study	
☒	☐	Antibodies
☒	☐	Eukaryotic cell lines
☒	☐	Palaeontology and archaeology
☐	☒	Animals and other organisms
☐	☒	Human research participants
☒	☐	Clinical data
☒	☐	Dual use research of concern

Methods

n/a	Involved in the study	
☒	☐	ChIP-seq
☒	☐	Flow cytometry
☒	☐	MRI-based neuroimaging

Animals and other organisms

Policy information about studies involving animals; ARRIVE guidelines recommended for reporting animal research

Laboratory animals	Mosquitoes (Aedes aegypti: Rockefeller, Liverpool, Gr3[ECFP]14 and opsin-1, opsin-2 mutant lines; Anopheles stephensi (Indian strain) and Culex quinquefasciatus) were raised at the University of Washington campus. Mosquito lines were provided from BEI Resources (Manassas, VA, USA) (Ae. aegypti: Rockefeller, Liverpool, Gr3[ECFP]; An. stephensi and Cx. quinquefasciatus). In the case of the Ae. aegypti opsin-1, opsin-2, and opsin-1,opsin-2 lines, we used existing mosquito lines that were generated in the Montell laboratory as previously described by Zhan et al. (2021).
Wild animals	*Provide details on animals observed in or captured in the field; report species, sex and age where possible. Describe how animals were caught and transported and what happened to captive animals after the study (if killed, explain why and describe method; if released, say where and when) OR state that the study did not involve wild animals.*
Field-collected samples	*For laboratory work with field-collected samples, describe all relevant parameters such as housing, maintenance, temperature, photoperiod and end-of-experiment protocol OR state that the study did not involve samples collected from the field.*
Ethics oversight	Because female mosquitoes are the disease vectors, we focus on females in these studies. Care and maintenance of the mosquito colonies are conducted within an Arthropod Containment Level 2 (ACL-2) facility and associated guidelines.

Note that full information on the approval of the study protocol must also be provided in the manuscript.

Human research participants

Policy information about studies involving human research participants

Population characteristics	3 males and 3 female individuals on the University of Washington (Seattle) campus (ages 25-46 years old).
Recruitment	Individuals were recruited from the campus at the University of Washington, in Seattle, WA, USA, and thus may not reflect the demographics of the PNW region, or the USA.
Ethics oversight	Protocols were reviewed and approved by the University of Washington Institutional Review Board, and all human volunteers gave their informed consent to participate in the research.

Note that full information on the approval of the study protocol must also be provided in the manuscript.
